# Foster Care and Health in Medicaid-Enrolled Children Experiencing Parental Opioid Use Disorder

**DOI:** 10.1001/jamanetworkopen.2024.10432

**Published:** 2024-05-08

**Authors:** Angélica Meinhofer, Nisha Chandra, Desislava Byanova, Katherine M. Keyes

**Affiliations:** 1 Department of Population Health Sciences, Weill Cornell Medicine, New York, New York; 2 Medical College, Weill Cornell Medicine, New York, New York; 3 Independent Researcher, Washington, DC; 4 Department of Psychiatry, Columbia University Medical Center/New York State Psychiatric Institute, New York

## Abstract

**Importance:**

The burden of the US opioid crisis has fallen heavily on children, a vulnerable population increasingly exposed to parental opioid use disorder (POUD) in utero or during childhood. A paucity of studies have investigated foster care involvement among those experiencing parental opioid use during childhood and the associated health and health care outcomes.

**Objective:**

To examine the health and health care outcomes of children experiencing POUD with and without foster care involvement.

**Design, Setting, and Participants:**

This population-based cohort study used nationwide Medicaid claims data from January 1, 2014, to December 31, 2020. Participants included Medicaid-enrolled children experiencing parental opioid use–related disorder during ages 4 to 18 years. Data were analyzed between January 2023 and February 2024.

**Exposure:**

Person-years with (exposed) and without (nonexposed) foster care involvement, identified using Medicaid eligibility, procedure, and diagnostic codes.

**Main Outcomes and Measures:**

The main outcomes included physical and mental health conditions, developmental disorders, substance use, and health care utilization. The Pearson χ^2^ test, the *t* test, and linear regression were used to compare outcomes in person-years with (exposed) and without (nonexposed) foster care involvement. An event study design was used to examine health care utilization patterns before and after foster care involvement.

**Results:**

In a longitudinal sample of 8 939 666 person-years from 1 985 180 Medicaid-enrolled children, 49% of children were females and 51% were males. Their mean (SD) age was 10 (4.2) years. The prevalence of foster care involvement was 3% (276 456 person-years), increasing from 1.5% in 2014 to 4.7% in 2020. Compared with those without foster care involvement (8 663 210 person-years), foster care involvement was associated with a higher prevalence of developmental delays (12% vs 7%), depression (10% vs 4%), trauma and stress (35% vs 7%), and substance use–related disorders (4% vs 1%; *P* < .001 for all). Foster children had higher rates of health care utilization across a wide array of preventive services, including well-child visits (64% vs 44%) and immunizations (41% vs 31%; *P* < .001 for all). Health care utilization increased sharply in the first year entering foster care but decreased as children exited care.

**Conclusions and Relevance:**

In this cohort study of Medicaid-enrolled children experiencing parental opioid use–related disorder, foster care involvement increased significantly between 2014 and 2020. Involvement was associated with increased rates of adverse health outcomes and health care utilization. These findings underscore the importance of policies that support children and families affected by opioid use disorder, as well as the systems that serve them.

## Key Points

**Question:**

Is foster care involvement associated with health and health care outcomes of Medicaid-enrolled children experiencing parental opioid use disorder?

**Findings:**

In this cohort study of 1 985 180 children experiencing parental opioid use disorder, foster care involvement increased from approximately 2% in 2014 to 5% in 2020. Compared with children without foster care involvement, foster children had significantly higher rates of mental health, developmental disorders, and substance use diagnoses and elevated levels of health care utilization.

**Meaning:**

These findings suggest that foster care involvement increased rapidly during the US opioid crisis and was associated with increased health care utilization and adverse health outcomes.

## Teaser

This cohort study assesses the health and health care outcomes of children experiencing parental opioid use disorder with and without foster care involvement.

## Introduction

Between 2000 and 2017, foster care entries involving parental drug use increased by 147.05% nationwide, whereas entries for other home removal reasons decreased during this period.^1^ This growth has been attributed to the US opioid crisis,^2,3,4^ which has increased the number of children experiencing parental opioid use disorder (POUD) in utero and during childhood. Between 2010 and 2017, the prevalence of neonatal abstinence syndrome, a condition of newborn withdrawal often resulting from prenatal opioid exposure, increased by 83%.^5^ Likewise, between 2002 and 2017, the number of children living with an adult who had an opioid pain reliever use disorder or used heroin increased by 30% and 200%, respectively.^6^

Parental opioid use disorder can adversely affect a child’s well-being.^7,8,9^ This disorder may contribute to unstable or harmful home environments, including child maltreatment, family separation due to parental overdose mortality, parental incarceration, and parental divorce, among other adverse childhood experiences.^10^ Such experiences may result in child welfare system involvement and subsequent foster care or other out-of-home placement. Children in foster care face greater risk of negative outcomes over the life course, either due to the maltreatment that precipitated the placement or by the disruption from the home removal.^11^ Adverse outcomes include acute and chronic illnesses, mental health conditions, and developmental and behavioral problems.^12,13,14,15,16^ Children in foster care also have higher rates of criminal justice system involvement, inadequate education, housing instability, and substance use disorder.^17,18,19,20,21^ The American Academy of Pediatrics considers children in foster care as having “special health care needs” due to their high rates of physical and mental health problems.^11^

Although the consequences of the opioid crisis on adults and newborns have received considerable attention, the outcomes associated with this crisis among children remain understudied.^7^ The increasing role of the foster care system in the lives of these children is also understudied. This study leveraged nationwide Medicaid claims from 2014 to 2020 to examine the health and health care outcomes of children experiencing POUD, with and without foster care involvement. We also used an event study design to examine the evolution of health care utilization, before and after the first year of foster care involvement.

## Methods

The Weill Cornell Medicine Institutional Review Board deemed this study exempt from review and waived the informed consent requirement because secondary data excluded direct identifiers. The study followed the Strengthening the Reporting of Observational Studies in Epidemiology (STROBE) reporting guideline.

### Data Source

We analyzed nationwide Medicaid claims from the 2014-2015 Medicaid Analytical eXtract (MAX) and the 2014-2020 Transformed Medicaid Statistical Information System Analytic Files (TAF) for January 1, 2014, to December 31, 2020. The MAX and TAF data capture claims information for all Medicaid enrollees in the 50 US states and the District of Columbia as well as for Children’s Health Insurance Program (CHIP) enrollees. Data files include demographic and eligibility files, inpatient hospital claims, other services claims, and prescription drug pharmacy claims. The demographic and eligibility files contain information on the demographics (eg, birth date, sex, zip code, and race and ethnicity), eligibility, and enrollment characteristics of beneficiaries enrolled in Medicaid or CHIP for at least 1 day in the calendar year. The beneficiary identifier (ID) identifies the same individual over time and across states. The state-assigned case ID identifies family units in most states.^22,23^ Claims files contain longitudinal service use (fee-for-service claims and managed care encounters) and payment records (eMethods 1 in Supplement 2).

### Study Population

We identified adults aged 19 years or older with an opioid use–related disorder (OUD) using diagnostic codes (*International Classification of Diseases, Ninth Revision* [*ICD-9*], or *International Statistical Classification of Diseases, Tenth Revision* [*ICD-10*]) indicating opioid abuse, dependence, or poisoning and using procedure codes or national drug codes indicating OUD medication treatment with methadone or buprenorphine.^24^ We linked adults with OUD to all other beneficiaries sharing the same case ID and zip code of residence at least once between 2014 and 2020.^22,23^ We excluded beneficiaries with a missing case ID, beneficiary ID, or date of birth. We removed inaccurate linkages and duplicate records. We selected children aged 4 to 18 years who were at least 18 years younger than the adult with OUD, and we imposed eligibility and enrollment criteria to increase the completeness of claims records. We excluded person-years with less than 30 days of enrollment, missing eligibility information, with separate state CHIP (S-CHIP) enrollment, with dual health insurance (ie, Medicare and Medicaid), and with restricted Medicaid benefits (eMethods 2 in Supplement 2).

### Measures

#### Foster Care Involvement

We generated an indicator variable identifying person-years in which a child had at least 1 month of foster care involvement.^25^ We defined foster care involvement using Medicaid eligibility codes (eMethods 3 in Supplement 2).^25^ Codes separately identified children with Title IV-E eligibility (labeled as “children with Title IV-E adoption assistance, foster care, or guardianship care”), children without Title IV-E eligibility but who received adoption assistance and had special medical needs (“children with non–IV-E adoption assistance”), former foster youth eligible through the Affordable Care Act (“former foster care children”), and foster youth eligible through the state Chafee Option (“independent foster care adolescents”).

The aforementioned eligibility codes will not identify foster children enrolled in Medicaid through other eligibility pathways (eg, low income, disability, pregnancy).^25^ This will primarily undercount foster children who do not meet Title IV-E requirements of being removed from a very low-income household.^26,27^ Previous work shows that despite nearly all foster children being enrolled in Medicaid, only about 73% of children in foster care for at least a year display a Medicaid code for foster care eligibility.^28^ We therefore supplemented Medicaid eligibility codes with diagnostic and procedure codes indicating foster care status, increasing person-years in care by 6 percentage points.

#### Child Measures

Measures of interest included demographics and Medicaid enrollment, physical and mental health conditions, developmental disorders, substance use–related disorders, and health care utilization. Measures were selected based on previous literature about foster care and Medicaid benefits under the Early and Periodic Screening, Diagnostic and Treatment program.^29^ We generated an indicator variable identifying person-years in which a child had at least 1 claim involving an outcome of interest.^25^ We identified outcomes in inpatient and outpatient claims using diagnostic and procedure codes (*ICD-9* and *ICD-10*; *Current Procedural Terminology, Fourth Edition*; and the *Healthcare Common Procedure Coding System*) and *Current Dental Terminology* codes (eMethods 1 and 4 in Supplement 2 and eTable 1 in Supplement 1).

Demographics included sex, race and ethnicity, and urbanicity. Race and ethnicity were identified in Medicaid data and were included to elucidate sociodemographic factors associated with foster care involvement. These data were reported as American Indian or Alaska Native, Hispanic, non-Hispanic Black (hereinafter, Black), non-Hispanic White (hereinafter, White), or non-Hispanic other race (hereinafter, other race; includes non-Hispanic Asian, non-Hispanic Native Hawaiian or Other Pacific Islander, or non-Hispanic multiple races).

Physical health conditions included acute and chronic conditions common in pediatric populations. Developmental conditions included autism and other pervasive developmental disorders as well as various cognitive, speech, and growth delays. Mental health conditions included depression, anxiety, trauma and stress disorders, attention-deficit/hyperactivity disorder (ADHD) and other conduct disorders, and suicidality (suicidal ideation, suicidal attempt, or self-harm). Substance use–related disorders included alcohol, tobacco, and drug abuse, dependence, or poisoning.

Health care services included well-child visits, immunizations, vision and hearing examinations, recommended laboratory tests, dental services, screening (developmental, mental health, and other health risk), mental health provider visits, health counseling, and visits in various health care settings (inpatient, emergency department, urgent care clinics, and school-based health services).

### Statistical Analysis

We structured the sample as a longitudinal dataset of person-years, in which each child could be observed for up to 7 years between 2014 and 2020 and their foster care status or outcomes could change during this time. We calculated time trends in the raw prevalence of foster care involvement across person-years. We then calculated and compared the raw prevalence of demographics and health outcomes across person-years, by foster care involvement. We used the Pearson χ^2^ test to compare categorical variables and the *t* test to compare continuous variables. We used linear regression to compute adjusted differences in outcome prevalence by foster care involvement, controlling for demographics, enrollment days, state and year fixed effects, and clustering SEs at the person level. Additionally, we stratified the sample into age groups (4-8, 9-13, and 14-18 years) to capture potential heterogeneity attributable to the natural stages of childhood development. We used the Cochran-Armitage linear trend test to verify whether outcome prevalence increased or decreased across age groups among the foster care group. *P* < .001 (2-tailed) was considered significant.

Finally, we used an event study design to examine the raw prevalence of foster care involvement and health care utilization, the years before and after the first year in foster care observed in the data (index year). We selected all foster children first entering foster care between ages 4 to 18 years and stratified the sample into groups using age at index year (4-8, 9-13, and 14-18 years). For each child, we required observation of the year immediately before the index year. We leveraged all person-years between ages 3 to 19 years for the children in our sample, which allowed us to observe up to 1 year before and after (preindex and postindex) for children entering care at ages 4 and 18 years, respectively (eMethods 5 and eTables 2 and 3 in Supplement 2). Data were analyzed with Stata, version 17 (StataCorp LLC) between January 2023 and February 2024.

## Results

**Figure 1.**
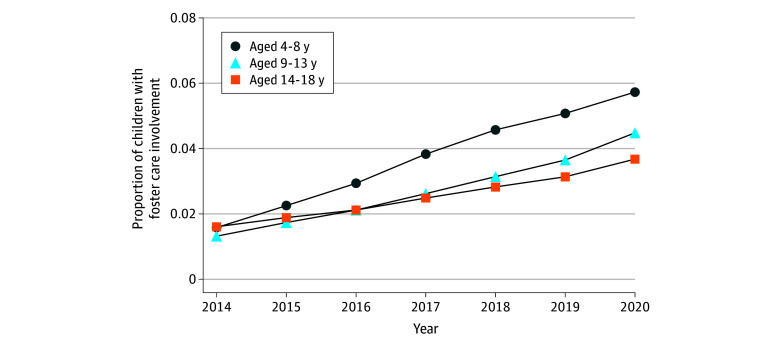
Trends in Foster Care Involvement Among Children Experiencing Parental Opioid Use–Related Disorder

Our longitudinal sample comprised 8 939 666 person-years from 1 985 180 Medicaid-enrolled children experiencing POUD who were aged 4 to 18 years between 2014 and 2020. Approximately 49% (4 390 712 person-years) were females and 51% (4 548 954 person-years) were males. Their mean (SD) age was 10 (4.2) years. For children with vs without foster care involvement, race and ethnicity was reported as American Indian or Alaska Native (5% vs 3%), Black (14% vs 15%), Hispanic (9% vs 14%), White (66% vs 61%), or other race (2% vs 2%; these data were missing for 4% vs 5%). Foster care involvement was identified in 3% of person-years (276 456 person-years from 125 009 children), increasing from 1.5% in 2014 to 4.7% in 2020 (213% increase). From 2014 to 2020, the prevalence of foster care involvement increased by 256% (from 1.6% to 5.7%), 246% (from 1.3% to 4.5%), and 131% (from 1.6% to 3.7%) among children aged 4 to 8, 9 to 13, and 14 to 18 years, respectively (Figure 1).

Table 1 reports demographic and enrollment characteristics of children experiencing POUD, by foster care involvement. Relative to children without foster care involvement (8 663 210 person-years), foster children (276 456 person-years) were more likely to be American Indian or Alaska Native (5% vs 3%) or White (66% vs 61%) and less likely to be from metropolitan areas (71% vs 75%; *P* < .001 for all, χ^2^ test). The mean (SD) number of days of Medicaid enrollment per year was quantitatively similar between groups (347 [57] vs 341 [66] days; *P* < .001, *t* test).

**Table 1.** Demographics and Medicaid Enrollment of Children Experiencing Parental Opioid Use–Related Disorder^a^

Characteristic	Age group, y							
	Foster care involvement^b^				No foster care involvement^b^			
	4-8	9-13	14-18	Total^c^	4-8	9-13	14-18	Total^c^
No. of PY	128 003	87 137	61 316	276 456	3 341 664	3 044 606	2 276 940	8 663 210
Sex, % of PY								
Female	48	49	50	49	49	49	49	49
Male	52	51	50	51	51	51	51	51
Race and ethnicity, % of PY^d^								
American Indian or Alaska Native	6	5	5	5	3	3	3	3
Hispanic	9	10	10	9	13	14	14	14
Non-Hispanic Black	12	14	17	14	15	15	16	15
Non-Hispanic White	66	66	63	66	61	62	60	61
Non-Hispanic other race^e^	2	2	1	2	2	2	2	2
Missing	5	4	4	4	6	5	5	5
Urbanicity, % of PY								
Metropolitan	70	71	73	71	75	75	76	75
Micropolitan	15	15	15	15	14	14	13	14
Small town	9	8	8	8	7	7	7	7
Rural	6	6	5	5	4	4	4	4
Medicaid enrollment, d, mean (SD)	346 (59)	349 (55)	348 (55)	347 (57)	341 (66)	342 (65)	340 (67)	341 (66)

Abbreviation: PY, person-years.

^a^ Medicaid claims were from the 2014-2020 Transformed Medicaid Statistical Information System Analytic Files and the 2014-2015 Medicaid Analytical eXtract. The unit of observation is person-years (N = 8 939 666) from 1 985 180 children (aged 4-18 years) experiencing parental opioid use disorder (identified with procedure codes, diagnostic codes, and national drug codes).

^b^ Sample of PY stratified by foster care involvement, identified using Medicaid eligibility, procedure, and diagnostic codes.

^c^ Pearson χ^2^ test used to compare categorical variables across PY with and without foster care involvement (total columns); *t* test used to compare continuous variables (enrollment days) (eTable 4 and eMethods 6 in Supplement 2). *P* < .001 for all variables except sex.

^d^ Identified in the Medicaid data and included to elucidate sociodemographic factors associated with foster care involvement.

^e^ Includes non-Hispanic Asian, non-Hispanic Native Hawaiian or Other Pacific Islander, or non-Hispanic multiple races.

Table 2 reports various health outcomes of children experiencing POUD, by foster care involvement. Both groups generally exhibited quantitatively similar physical health outcomes, although injuries were notably elevated at older ages (14-18 years: 29% vs 21%; *P* < .001, χ^2^ test). Foster children had higher rates of developmental delays (12% vs 7%; *P* < .001, χ^2^ test), although prevalence decreased at older ages. Foster children also exhibited higher rates of mental health conditions, with elevated prevalence at older ages. This included higher rates of depression (10% vs 4%), anxiety (9% vs 5%), trauma and stress (35% vs 7%), and ADHD, conduct disorder, or impulse disorder (25% vs 12%; *P* < .001 for all, χ^2^ test). Notably, foster children had higher rates of substance use–related disorders at older ages (14-18 years: 15% vs 4%; *P* < .001, χ^2^ test).

**Table 2.** Health Diagnoses of Children Experiencing Parental Opioid Use–Related Disorder^a^

Characteristic	Age group, y								Mean difference (95% CI)^f^
	Foster care involvement^b^^,^^c^				No foster care involvement^b^				
	4-8	9-13	14-18^d^	Total^e^	4-8	9-13	14-18^d^	Total^e^	
No. of PY	128 003	87 137	61 316	276 456	3 341 664	3 044 606	2 276 940	8 663 210	8 939 666
Physical health, % of PY									
Congenital abnormality	4	2	2	3	2	2	2	2	0.98 (0.90-1.06)
Asthma	8	7	8	8	8	8	7	8	0.39 (0.25-0.52)
Obesity	1	4	5	3	2	4	4	3	0.40 (0.33-0.48)
Hearing problem	3	2	1	2	2	1	1	1	0.92 (0.86-0.99)
Vision problem	4	3	3	3	2	2	2	2	1.35 (1.27-1.43)
Middle ear infection	20	8	5	13	18	8	5	11	1.19 (1.05-1.33)
Respiratory infection	42	31	29	36	41	32	29	35	0.72 (0.51-0.93)
Dental problem	9	6	6	7	6	4	3	5	2.16 (2.06-2.27)
Dermatologic problem	15	13	18	15	14	12	15	13	2.19 (2.04-2.34)
Injuries	16	19	29	20	15	18	21	18	3.45 (3.29-3.62)
Complex chronic condition	4	5	10	6	4	5	7	5	1.67 (1.56-1.79)
Developmental disorder, % of PY									
Autism or PDD	3	2	2	2	2	1	1	1	0.97 (0.88-1.05)
Developmental delay	18	10	5	12	9	7	3	7	4.91 (4.74-5.07)
Mental health disorder, % of PY									
Depression	1	9	27	10	0	3	11	4	6.05 (5.91-6.19)
Anxiety	5	10	18	9	2	4	9	5	4.84 (4.71-4.98)
Trauma and stress	30	40	35	35	5	8	8	7	27.4 (27.2-27.6)
ADHD, conduct disorder, or impulse	17	31	33	25	8	16	13	12	14.0 (13.7-14.2)
Suicidality or self-harm	0	3	8	3	0	1	2	1	2.05 (1.98-2.13)
SUD, % of PY	0	1	15	4	0	0	4	1	2.96 (2.88-3.05)
Alcohol	0	0	3	1	0	0	1	0	0.62 (0.58-0.66)
Tobacco	0	0	3	1	0	0	1	0	0.57 (0.53-0.61)
Drug	0	1	13	3	0	0	3	1	2.55 (2.47-2.63)

Abbreviations: ADHD, attention-deficit/hyperactivity disorder; PDD, pervasive developmental disorder; PY, person-years; SUD, substance use–related disorder.

^a^ Medicaid claims from the 2014-2020 Transformed Medicaid Statistical Information System Analytic Files and the 2014-2015 Medicaid Analytical eXtract. The unit of observation is PY (N = 8 939 666) from 1 985 180 children (aged 4-18 years) experiencing parental opioid use disorder (identified with procedure codes, diagnostic codes, and national drug codes).

^b^ Sample of PY stratified by foster care involvement, identified using Medicaid eligibility, procedure, and diagnostic codes.

^c^ Cochran-Armitage linear trend used to test whether outcomes increased or decreased across age in the foster care group (eTable 4 and eMethods 6 in Supplement 2). *P* < .001 for all variables.

^d^ Pearson χ^2^ test used to compare categorical variables across PY with and without foster care involvement (ages 14-18 years). *P* < .001 for all variables except middle ear infection.

^e^ Pearson χ^2^ test used to compare categorical variables across PY with and without foster care involvement (total columns) (eTable 4 and eMethods 6 in Supplement 2). *P* < .001 for all variables except obesity.

^f^ Linear regression used to compute adjusted differences in outcome prevalence across PY with and without foster care involvement (total columns). Regressions controlled for demographics and enrollment days in Table 1 and for state and year fixed effects. Standard errors were clustered at the person level. *P* < .001 for all variables. Adjusted differences for each age group are in eTable 6 and eMethods 6 in Supplement 2.

Table 3 reports health care outcomes for children experiencing POUD by foster care involvement. Foster children had higher levels of health care utilization across most measures, with notable differences for well-child visits (64% vs 44%), immunizations (41% vs 31%), laboratory work (34% vs 25%), dental services (67% vs 49%), and mental health provider visits (44% vs 14%; *P* < .001 for all, χ^2^ test). Foster children also had higher rates of inpatient hospital visits (12% vs 7%) and school-based health services (18% vs 12%; *P* < .001 for all, χ^2^ test).

**Table 3.** Health Care Utilization of Children Experiencing Parental Opioid Use–Related Disorder^a^

Characteristic	Age group, y								Mean difference (95% CI)^f^
	Foster care involvement^b^^,^^c^				No foster care involvement^b^				
	4-8	9-13	14-18^d^	Total^e^	4-8	9-13	14-18^d^	Total^e^	
No. of PY	128 003	87 137	61 316	276 456	3 341 664	3 044 606	2 276 940	8 663 210	8 939 666
Primary care and prevention, % of PY									
Well-child visit	69	62	57	64	52	42	38	44	20.61 (20.40-20.81)
Immunization	44	39	37	41	33	31	28	31	10.85 (10.65-11.05)
Vision examination	22	23	20	22	19	18	15	18	6.10 (5.93-6.28)
Hearing examination	23	21	17	21	19	15	11	15	7.32 (7.14-7.49)
Laboratory work	32	29	46	34	25	21	30	25	10.16 (9.95-10.37)
Developmental screen	13	6	5	9	8	3	3	5	2.91 (2.80-3.03)
Metabolic screen	2	9	15	7	2	6	8	5	3.24 (3.13-3.36)
Lead screen	11	1	1	6	7	0	0	3	2.64 (2.55-2.73)
STI screen	2	2	20	6	1	1	9	3	3.63 (3.53-3.74)
Health counseling	10	9	10	10	6	5	5	5	3.90 (3.77-4.02)
Dental services, % of PY	69	69	63	67	51	51	43	49	19.06 (18.85-19.27)
Mental health services, % of PY									
Mental health screen	12	19	22	16	3	7	10	6	9.98 (9.82-10.15)
Mental health provider visit	36	49	53	44	9	15	17	14	29.95 (29.71-30.19)
Other health care settings, % of PY									
Inpatient hospital	9	11	17	12	7	6	9	7	4.05 (3.92-4.18)
Emergency department	30	28	42	32	32	27	32	30	3.53 (3.32-3.73)
Urgent care clinic	13	11	12	12	11	10	11	11	0.70 (0.55-0.84)
School-based health services	17	20	15	18	12	14	9	12	6.79 (6.61-6.98)

Abbreviations: PY, person-years; STI, sexually transmitted infection.

^a^ Medicaid claims from the 2014-2020 Transformed Medicaid Statistical Information System Analytic Files and the 2014-2015 Medicaid Analytical eXtract. The unit of observation is PY (N =8 939 666) from 1 985 180 children (ages 4-18 years) experiencing parental opioid use disorder (identified with procedure codes, diagnostic codes, and national drug codes).

^b^ Sample of PY stratified by foster care involvement, identified using Medicaid eligibility, procedure, and diagnostic codes.

^c^ Cochran-Armitage linear trend test used to verify whether outcomes increased or decreased across age groups in the foster care group (eTable 5 and eMethods 6 in Supplement 2). *P* < .001 for all variables except health counseling.

^d^ Pearson χ^2^ test used to compare categorical variables across PY with and without foster care involvement (ages 14-18 years). *P* < .001 for all variables.

^e^ Pearson χ^2^ test used to compare categorical variables across PY with and without foster care involvement (total columns) (eTable 5 and eMethods 6 in Supplement 2). *P* < .001 for all variables.

^f^ Linear regression used to compute adjusted differences in outcome prevalence across PY with and without foster care involvement (total columns). Regressions controlled for demographics and enrollment days in Table 1 and for state and year fixed effects. Standard errors were clustered at the person level. Adjusted differences for each age group are in eTable 7 and eMethods 6 in Supplement 2.

**Figure 2.**
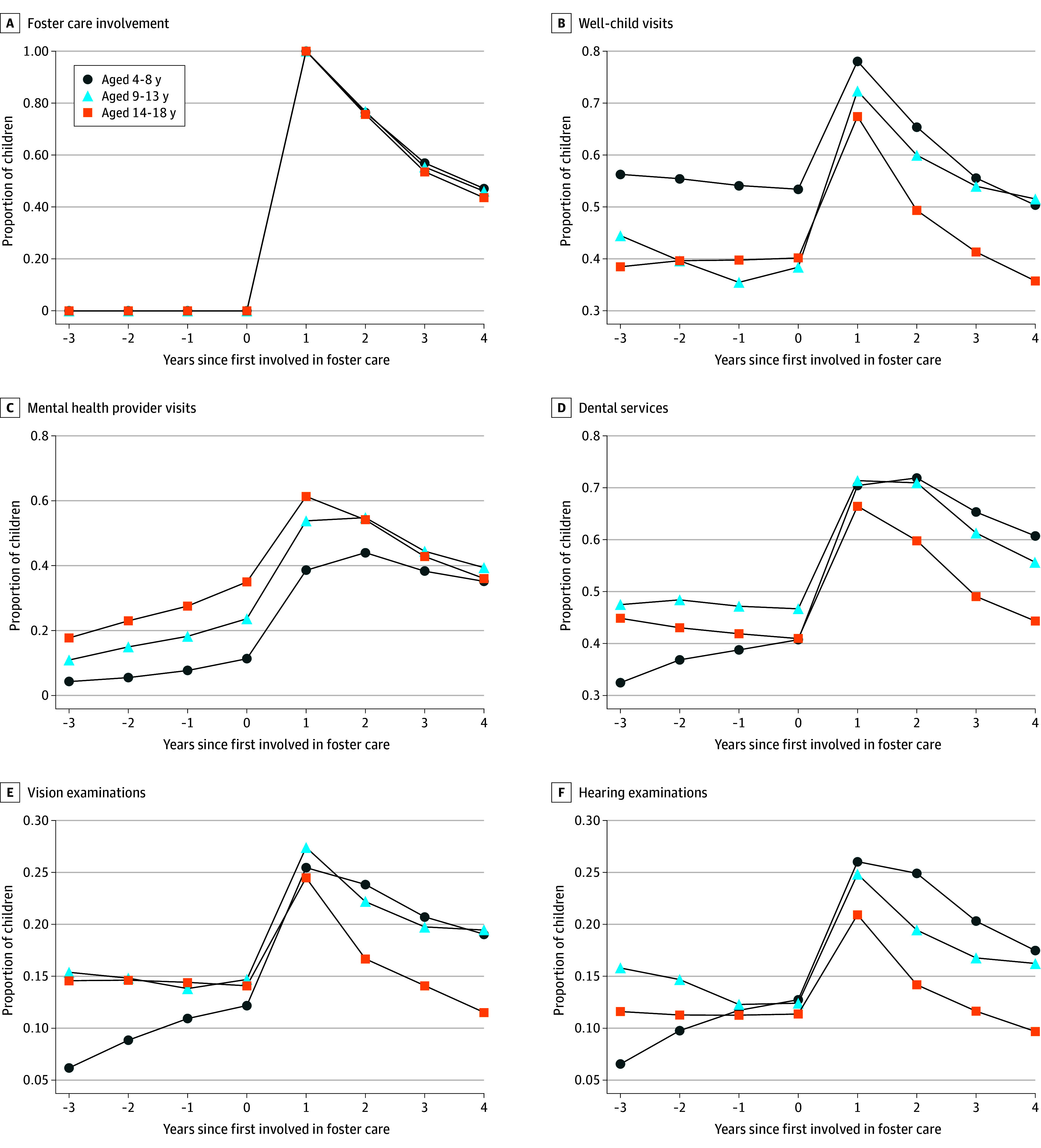
Health Care Utilization of Children Experiencing Parental Opioid Use–Related Disorder, Since First Year of Foster Care Involvement A, Evolution of foster care involvement for children experiencing parental opioid use–related disorder who first became involved during the study period. Year 1 denotes the index year, when a child entered foster care. Most children remained in foster care for less than 4 years, irrespective of their age at entry. B to F, Proportion of children utilizing certain health care services before and after their initial year in foster care, including well-child visits (B), mental health provider visits (C), dental services (D), vision examinations (E), and hearing examinations (F).

Finally, Figure 2A depicts the evolution of foster care involvement for children experiencing POUD who first became involved during the study period. Year 1 denotes the index year, when a child entered foster care. Most children remained in foster care for less than 4 years, irrespective of their age at entry. Figure 2B to F depicts the proportions of these children who utilized certain health care services before and after their initial year in foster care. Across all age groups, we observed sharp increases in health care utilization during the first year in care, followed by decreases in subsequent years. This pattern closely mirrored the evolution of foster care involvement, suggesting that health care utilization increased considerably while in foster care but decreased after exit.

## Discussion

Foster care involvement can have profound and multifaceted implications on a child’s life. Although foster care placement may offer a safe haven from potentially harmful or unstable home environments, this life disruption may also lead to a host of psychological challenges and affect development and health.^30^ This study documents that foster care involvement increased rapidly among Medicaid-enrolled children experiencing POUD, from 1.5% in 2014 to 4.7% in 2020 (213% increase). In contrast, the total number of children served by the foster care system changed modestly during this time, from 646 000 in 2014 to 632 000 in 2020 (−2% decrease).^31^ Together, our findings suggest that children experiencing POUD are becoming involved with the foster care system at faster rates than children in the general population.

In this study, American Indian or Alaska Native children were disproportionately represented among children experiencing POUD (3%) and in foster care (5%), despite representing 1% of the US child population.^32^ Previous literature is consistent with these findings, showing that American Indian or Alaska Native children display the highest rate of foster care entries due to parental drug use.^33^

Foster children in this study had higher rates of mental health, developmental, and substance use diagnoses relative to those not involved in foster care. This reinforces findings in previous studies showing that foster children exhibit higher rates for adverse outcomes in all of the above domains.^10,11,12,13,14,15,16,17,18,19,20,21,34,35^ Our study demonstrates that these disparities persist within the population of children experiencing POUD.

We also found increased health care utilization among foster children. This included greater utilization of preventive and primary care services that are recommended during childhood, irrespective of POUD exposure, as well as an elevated number of inpatient hospital visits and greater usage of school-based health services. Increases in health care utilization occurred immediately after foster care entry and decreased as children exited care. This finding suggests that children are more likely to receive health care services while in foster care, but cease to receive these services after exiting care and are less likely to receive them prior to entering care.

The implications of elevated health diagnoses, coupled with increased health care utilization among foster children, are likely multifaceted with at least 3 potential explanations. First, children experiencing POUD placed in foster care likely experienced maltreatment and may have associated health conditions and unmet health care needs prior to their placement. Second, foster care placement is a traumatic experience in itself and could be a contributing factor for the elevated rates of health conditions among foster children.^11^ Third, our results could be explained by federal requirements that state child welfare agencies face for health screening and assessment of children as soon as they enter foster care.^35,36^ Increased health surveillance is one way in which the welfare system supports foster children. The resulting higher rates of health diagnoses among foster children may be simply a by-product of heightened health monitoring and may mask the true incidence of health issues among the broader population of children experiencing POUD. That is, children experiencing POUD who remain uninvolved with the foster care system may exhibit similar health care needs to their foster-involved counterparts but are undertreated and thus underdiagnosed. This could result from impaired parenting capacity or resources, which have been associated with parental drug use in previous research.^37,38^

Although further research is needed to disentangle the mechanisms at play, our study findings suggest that children experiencing POUD are a vulnerable population with increasing foster care involvement and the need for regular and reliable health evaluation and provision of adequate health care, irrespective of their foster care involvement. Policy makers and clinicians must ensure that the unique health care needs of children experiencing POUD are met with early identification, prevention, and treatment. Families affected by OUD should not be punished but supported in remaining together when safe and appropriate through OUD treatment and ancillary services, as parents who receive these services are more likely to return to their parental roles.^39^ The outcomes of foster children will depend on several factors, 2 of which include the commitment of foster parents and child welfare agencies. Therefore, increasing support and resources for child welfare agencies and foster parents is crucial. Although local child welfare agencies remain the primary response for children affected by the opioid crisis,^40^ these agencies continue to face substantial challenges, including high caseloads, foster parent shortages, and caseworker burnout.^41^

### Strengths and Limitations

This study contributes to the existing literature in several ways. First, we focused on children experiencing POUD, a fast-growing yet understudied population with complex family environments, increased risk of maltreatment, and special health care needs. Second, we estimated the prevalence of foster care involvement and whether outcomes differ based on this exposure. Third, we analyzed Medicaid claims, which are detailed, longitudinal, and nationwide. Importantly, Medicaid is a critical source of health insurance for children in the welfare system,^42,43^ covering about 99% of children in foster care^44,45^ and 85% of children who aged out of foster care.^46^ We were therefore able to produce generalizable estimates for a comprehensive set of individual-level outcomes. This overcomes limitations in previous studies, which either rely on data from small or single-state samples, self-reported measures, or ecological designs or whose study period precedes the opioid crisis. The few population-level studies analyzed commonly used child welfare datasets, which include a limited number of underreported child health outcomes and do not indicate the drug used by the parent. Results from this timely study can help inform policies that support children and families affected by the US opioid crisis, as well as the systems that serve them.

This study is not without limitations. First, Medicaid eligibility codes do not capture all children in foster care. This measure primarily undercounts Medicaid-enrolled foster children who do not meet Title IV-E requirements. Nevertheless, our measure captures some of the most vulnerable children served by the foster care system. Second, we are unable to ascertain whether increases in foster care placement resulted from greater maltreatment or greater stigma against parents with OUD. Third, the case ID does not identify families in 5 states (eMethods 2 in Supplement 2). Moreover, Medicaid data do not identify familial relationships between beneficiaries sharing a case ID; therefore, our approximation using age may not always correctly identify parents. Finally, the extent to which the patterns we observed over time or across age groups reflect a combination of age, period, and cohort effects is unresolved.

## Conclusions

In this cohort study of Medicaid-enrolled children experiencing POUD, we observed that foster care involvement increased considerably between 2014 and 2020. Foster care involvement was associated with worse mental health, developmental, and substance use–related outcomes and with higher health care utilization.

## Notes

Supplement 1. **eTable 1. **Codes Used

Supplement 2. **eMethods 1. **Terminology Specific to the US Health Care System **eMethods 2. **Study Population **eMethods 3. **Medicaid Coverage of Children in the Foster Care System **eMethods 4. **Outcome Codes **eMethods 5. **Event Study Plots **eTable 2. **Total Number of Person-Years in Event Study Plots **eTable 3. **Number of Person-Years With Foster Care Involvement in Event Study Plots **eMethods 6. **Results From Statistical Tests and Other Analyses **eTable 4. **Results From Statistical Tests Applied to Tables 1 and 2 **eTable 5. **Results From Statistical Tests Applied to Table 3 **eTable 6. **Mean Differences in Health Diagnoses by Foster Care Status **eTable 7. **Mean Differences in Health Care Utilization by Foster Care Status
eReferences

Supplement 3.
Data Sharing Statement
