# Supplementary material for: Foster Care and Health in Medicaid-Enrolled Children Experiencing Parental Opioid Use Disorder
Source: JAMA Netw Open. 2024 May 8;7(5):e2410432. doi: 10.1001/jamanetworkopen.2024.10432 (PMC11079692; doi:10.1001/jamanetworkopen.2024.10432)
Supplement: Supplement 2. — eMethods 1. Terminology Specific to the US Health Care System eMethods 2. Study Population eMethods 3. Medicaid Coverage of Children in the Foster Care System eMethods 4. Outcome Codes eMethods 5. Event Study Plots eTable 2. Total Number of Person-Years in Event Study Plots eTable 3. Number of Person-Years With Foster Care Involvement in Event Study Plots eMethods 6. Results From Statistical Tests and Other Analyses eTable 4. Results From Statistical Tests Applied to Tables 1 and 2 eTable 5. Results From Statistical Tests Applied to Table 3 eTable 6. Mean Differences in Health Diagnoses by Foster Care Status eTable 7. Mean Differences in Health Care Utilization by Foster Care Status eReferences [file jamanetwopen-e2410432-s002.pdf]

Supplementary Online Content

Meinhofer A, Chandra N, Byanova D, Keyes K. Foster care and health in Medicaid-enrolled children experiencing parental opioid use disorder. *JAMA Netw Open*. 2024;7(5):e2410432. doi:10.1001/jamanetworkopen.2024.10432

eMethods 1. Terminology Specific to the US Health Care System

eMethods 2. Study Population

eMethods 3. Medicaid Coverage of Children in the Foster Care System

eMethods 4. Outcome Codes

eMethods 5. Event Study Plots

eTable 2. Total Number of Person-Years in Event Study Plots

eTable 3. Number of Person-Years With Foster Care Involvement in Event Study Plots

eMethods 6. Results From Statistical Tests and Other Analyses

eTable 4. Results From Statistical Tests Applied to Tables 1 and 2

eTable 5. Results From Statistical Tests Applied to Table 3

eTable 6. Mean Differences in Health Diagnoses by Foster Care Status

eTable 7. Mean Differences in Health Care Utilization by Foster Care Status

eReferences

This supplementary material has been provided by the authors to give readers additional information about their work.

© 2024 Meinhofer A et al. *JAMA Network Open.*

eMethods 1. Terminology Specific to the US Health Care System

Below we define a number of terms used in our manuscript.

i. **Medicaid** is a joint federal and state program that helps cover medical costs for eligible people with limited income and resources.

ii. **Medicare** is federal health insurance for people 65 or older, and some people under 65 with certain disabilities or conditions.

iii. **Dual health insurance** refers to instances when people have both Medicare and full Medicaid coverage.

iv. **The Children's Health Insurance Program** (**CHIP**) provides health insurance coverage to children whose families are unable to obtain employer-sponsored insurance or purchase private non-group coverage but whose incomes are above the limits that would qualify their children for Medicaid. States have the option to set up a separate state program (**S-CHIP**) that offers a different package of benefits from CHIP.

v. **Fee-for-service** is a method in which doctors and other health care providers are paid for each service performed.

vi. **Managed care** is a health care delivery system organized to manage cost, utilization, and quality.

vii. The International Classification of Diseases, Ninth Revision (**ICD-9**) is an official system of assigning codes to diagnoses and procedures associated with hospital utilization in the United States. The ICD-9 was used until 2015, when use of **ICD-10** coding started.

viii. **The Current Procedural Terminology** (**CPT**) coding system is used for coding and describing healthcare services and procedures in federal programs (Medicare and

© 2024 Meinhofer A et al. *JAMA Network Open.*

Medicaid) and throughout the United States by private insurers and providers of healthcare services.

ix. **The Current Dental Terminology** (**CDT**) codes are procedural codes for oral health and adjunctive services provided in dentistry.

x. **The Healthcare Common Procedure Coding System** (**HCPCS**) is a collection of standardized codes that represent medical procedures, supplies, products, and services, which are used to facilitate the processing of health insurance claims.

xi. **Well-child visits** are routine doctor visits for comprehensive preventive health services that occur when a baby is young and annual visits until a child reaches age 21. Services include physical exam and measurements, vision and hearing screening, and oral health risk assessments.

eMethods 2. Study Population

Additional sample selection criteria are clarified in what follows. Some beneficiaries were assigned more than one Case ID during the study period. We used all Case IDs ever assigned to adult beneficiaries with OUD to link these beneficiaries with all other beneficiaries sharing the same Case ID and ZIP code at least once between 2014 and 2020. In five states (CT, NJ, WA, TX, NC), a large share of Case IDs did not match to beneficiaries other than the adult beneficiary with OUD. Therefore, our sample does not capture children in those states except in a small number of cases where a child moved to one of those states but was linked to an adult with OUD while living in a different state. In fewer instances, a small number of Case IDs matched to an unreasonably high number of beneficiaries. We dropped Case IDs associated with a single beneficiary or with more than 15 beneficiaries as these would likely not reflect family units. We required that the Case ID included at least one child aged 18 or younger and at least one adult

© 2024 Meinhofer A et al. *JAMA Network Open.*

with OUD aged 19 or older. We required the adult with OUD to be at least 18 years older than the child and to have the same ZIP code of residence as the child for at least one year between 2014 and 2020. We identified 2,441,203 unique children ages 0-18 meeting the previously described selection criteria imposed on adults with OUD or Case ID. We then imposed the following continuous enrollment and eligibility criteria on children following previous studies and definitions in the Centers for Medicare & Medicaid Services TAF Technical Guide: We dropped person-years with less than 30 days of enrollment; without comprehensive benefits for all months of enrollment (e.g., family planning only, or care limited to a specific condition); missing eligibility information for all months of enrollment; enrolled in S-CHIP for all months of enrollment; or with dual health insurance (i.e., Medicare and Medicaid) for all months of enrollment. This reduced our sample to 2,307,315 children ages 0-18. Lastly, we dropped person-years in which a child was ages 0 to 3, further reducing our sample to 1,985,180 children ages 4-18 between 2014 and 2020. We reincorporated person-years 3 and 19 into the sample for event study analyses.

eMethods 3. Medicaid Coverage of Children in the Foster Care System

About 99% of all children currently in foster care are covered by Medicaid through various means of eligibility.[1,2] Many children in foster care are categorically eligible for Medicaid through Title IV-E of the Social Security Act, which provides federal funding to support children in foster care, as well as children requiring adoption and guardianship assistance.[3] However, less than half of all children in foster care are Title IV-E eligible due to the policy's stringent eligibility requirements.[4] This includes the requirement that the child was removed from an extremely low-income household, as well as requirements surrounding how and why the child

© 2024 Meinhofer A et al. *JAMA Network Open.*

was removed from the home, the age and citizenship status of the child, and the placement setting and foster care provider for the child.[4]

Almost all non-IV-E eligible current foster care children are still eligible for Medicaid through various state-dependent pathways, most commonly through low-income pathways.[3] Non-IV-E eligible children requiring adoption or guardianship assistance may also still be eligible for Medicaid through state-dependent pathways, such as disability pathways.[3]

Certain children and young adults who were previously in foster care may also be Medicaid eligible.[3] Under the Patient Protection and Affordable Care Act, states are required to maintain Medicaid coverage of youth under age 26 who are no longer in foster care and were previously receiving Medicaid (either through Title IV-E or non-Title IV-E). Under the Chafee Option of the Social Security Act, states may also choose to cover other former foster care children up to age 21 without requiring that they had prior Medicaid enrollment or were in foster care in the same state in which the youth currently resides.

eMethods 4. Outcome Codes

Health outcomes were recorded using the ICD-9, ICD-10, CPT, HCPCS, and CDT codes listed in Supplementary Information eTable 1, attached separately. Outcomes were defined and grouped based on existing literature. We prioritized codes from validation studies when available.

Physical health outcomes were defined using codes for asthma,[5,6] congenital anomalies,[7,8] complex chronic conditions,[9] obesity,[10,11] hearing problems,[12,13] vision problems,[14,15] dental problems,[16,17] dermatologic problems,[18,19] middle ear infections,[20,21] respiratory infections,[22,23] gastroenteritis,[24,25] and injuries.[26]

© 2024 Meinhofer A et al. *JAMA Network Open.*

Mental health outcomes were defined using codes for depression,[27-29] anxiety,[27,30,31] suicidality (including suicidal ideation and suicide attempts) and self-harm,[32,33] trauma and stress disorders (including PTSD, adjustment reaction, and other reactions to stress),[34-36] ADHD/conduct/impulse disorders,[37-40] and other childhood emotional and social disorders (including attachment disorder, separation anxiety disorder, and other emotional/social disorders with onset usually occurring in childhood).[41-44]

Developmental outcomes were defined using codes for pervasive developmental disorders (including Autism, Asperger's, and unspecified disorders),[45-47] and developmental delays in speech/language, cognition, motor function, growth, and other domains.[41,48,49] Substance use outcomes were defined using codes for alcohol use, tobacco use, cannabis use, opioid use, stimulant use, and other drug use disorders.[27]

Healthcare utilization was defined using codes for pediatric preventative care included in the Medicaid Early and Periodic Screening, Diagnostic, and Treatment program,[50] including well-child visits, other outpatient visits, pediatric vaccines, vision exams, hearing exams, general dental services (which we supplemented with type of service codes from the Medicaid claims data), dental exams, fluoride administration, developmental screening, mental health screening, mental health provider visits, substance use screening, other health risk screening, health counseling, and recommended laboratory tests. Measures of health service settings were generated using Medicaid coding guidance, and included inpatient visits, emergency room visits, urgent care clinic visits, and school-based service visits.

© 2024 Meinhofer A et al. *JAMA Network Open.*

eMethods 5. Event Study Plots

eTable 2 reports the number of person-years for each event period by age group cell, which reflect the denominators in event study plots in Figure 2. eTable 3 reports the number of person-years with foster care involvement for each event period by age group cell, which reflect the numerators in event study plots in the top left panel of Figure 2.

eTable 2. Total Number of Person-Years in Event Study Plots

Year since first involved in foster care	*Age categories*			
	Ages 4-8	***Ages 9-13***	***Ages 14-18***	***Total***
-3	6,482	11,994	8,048	26,524
-2	13,588	15,799	10,612	39,999
-1	24,077	19,823	13,198	57,098
0	38,944	23,915	16,089	78,948
1	38,944	23,915	16,089	78,948
2	31,178	18,782	12,402	62,362
3	23,754	13,985	8,042	45,781
4	16,403	9,604	4,304	30,311
Total	193,370	137,817	88,784	419,971

eTable 3. Number of Person-Years With Foster Care Involvement in Event Study Plots

Year since first involved in foster care	*Age categories*			
	Ages 4-8	***Ages 9-13***	***Ages 14-18***	***Total***
1	38,944	23,915	16,089	78,948
2	23,797	14,418	9,379	47,594
3	13,526	7,743	4,307	25,576
4	7,728	4,424	1,874	14,026
Total	83,995	50,500	31,649	166,144

© 2024 Meinhofer A et al. *JAMA Network Open.*

eMethods 6. Results From Statistical Tests and Other Analyses

The below exhibits report main results from statistical tests applied to the variables displayed in Tables 1, 2, and 3. eTable 4 and eTable 5 report results from (1) the Pearson's X^2 test for comparison of categorical variables across group totals (i.e., with and without foster care involvement ages 4-18); (2) the t- test for comparison of continuous variables across group totals (i.e., with and without foster care involvement ages 4-18), and (3) the Cochran-Armitage test for linear trends across age groups (4-8, 9-13, 14-18) among the foster care group. eTable 4 displays results associated with demographics, Medicaid enrollment, and health diagnoses while eTable 5 displays results for healthcare utilization. Lastly, eTables 6 and 7 report adjusted mean differences by age group, applied to the variables displayed in Tables 2 and 3, respectively.

eTable 4. Results From Statistical Tests Applied to Tables 1 and 2[a]

	P-values for Pearson's X^2 test / t-test between group totals [b]	Cochran-Armitage test for trends across age groups in the foster care group [b]	
		Increase / Decrease	P-value
Demographics			
Sex	0.006		
Race and Ethnicity [c]	0.000		
Urbanicity [c]	0.000		
Medicaid enrollment days	0.000		
Physical health			
Congenital abnormality	0.000	Decrease	0.000
Asthma	0.000	Increase	0.000
Obesity	0.002	Increase	0.000
Hearing problem	0.000	Decrease	0.000
Vision problem	0.000	Decrease	0.000
Middle ear infection	0.000	Decrease	0.000
Respiratory infection	0.000	Decrease	0.000
Dental problem	0.000	Decrease	0.000
Dermatologic problem	0.000	Increase	0.000
Injuries	0.000	Increase	0.000
Complex chronic condition	0.000	Increase	0.000
Developmental disorder			
Autism/PDD	0.000	Decrease	0.000
Developmental delay	0.000	Decrease	0.000
Mental health disorder			
Depression	0.000	Increase	0.000
Anxiety	0.000	Increase	0.000
Trauma and Stress	0.000	Increase	0.000
ADHD/Conduct/Impulse	0.000	Increase	0.000
Suicidality/Self-Harm	0.000	Increase	0.000
Substance use disorder	0.000	Increase	0.000
Alcohol	0.000	Increase	0.000
Tobacco	0.000	Increase	0.000
Drug	0.000	Increase	0.000

Notes: Abbreviations: PDD, pervasive developmental disorder; ADHD, attention-deficit/hyperactivity disorder

[a] Medicaid claims data from the 2014-2020 Transformed Medicaid Statistical Information System Analytic Files (TAF) and the 2014-2015 Medicaid Analytical eXtract (MAX). The unit of observation are person-years (N = 8,939,666), from 1,985,180 children aged 4-18 experiencing POUD (identified with procedure, diagnostic, and NDC codes).

[b] The sample of person-years was stratified by foster care involvement (foster care status was identified using Medicaid eligibility, procedure and diagnostic codes). The Pearson's X^2 test was used to compare categorical variables across person-years with and without foster care involvement (total columns in Tables 1 and 2), while the t-test was used to compare continuous variables (enrollment days). The Cochran-Armitage test was used to test for linear trends across age groups (4-8, 9-13, 14-18) among the foster care group.

[c] Race and ethnicity categories included the following: black, non-Hispanic; Hispanic; Native American, non-Hispanic; Other race, non-Hispanic; white, non-Hispanic; and missing race. Urbanicity categories included the following: metropolitan, micropolitan, small town, and rural.

© 2024 Meinhofer A et al. *JAMA Network Open.*

eTable 5. Results From Statistical Tests Applied to Table 3[a]

	P-values for Pearson's X^2 test between group totals [b]	Cochran-Armitage test for trends across age groups in the foster care group [b]	
		Increase / Decrease	P-value
Primary Care and Prevention			
Well-Child Visit	0.000	Decrease	0.000
Immunization	0.000	Decrease	0.000
Vision Exam	0.000	Decrease	0.000
Hearing Exam	0.000	Decrease	0.000
Laboratory Work	0.000	Increase	0.000
Developmental Screen	0.000	Decrease	0.000
Metabolic Screen	0.000	Increase	0.000
Lead Screen	0.000	Decrease	0.000
STI Screen	0.000	Increase	0.000
Health Counseling	0.000	--	0.313
Dental Services	0.000	Decrease	0.000
Behavioral Services			
Mental Health Screen	0.000	Increase	0.000
Mental Health Provider	0.000	Increase	0.000
Other Healthcare Settings			
Inpatient Hospital	0.000	Increase	0.000
Emergency Room	0.000	Increase	0.000
Urgent Care Clinic	0.000	Decrease	0.000
School-Based Services	0.000	Decrease	0.000

Notes: Abbreviations: STI, sexually transmitted infection

[a] Medicaid claims data from the 2014-2020 Transformed Medicaid Statistical Information System Analytic Files (TAF) and the 2014-2015 Medicaid Analytical eXtract (MAX). The unit of observation are person-years (N = 8,939,666), from 1,985,180 children aged 4-18 experiencing POUD (identified with procedure, diagnostic, and NDC codes).

[b] The sample of person-years was stratified by foster care involvement (foster care status was identified using Medicaid eligibility, procedure and diagnostic codes). The Pearson's X^2 test was used to compare categorical variables across person-years with and without foster care involvement (total columns in Tables 3). The Cochran-Armitage test was used to test for linear trends across age groups (4-8, 9-13, 14-18) among the foster care group.

eTable 6. Mean Differences in Health Diagnoses by Foster Care Status[a,b,c]

	Adjusted Mean Differences [95% CI]			
Age group	4 to 8	9 to 13	14 to 18	Total
Person Years (PY)	3,469,667	3,131,743	2,338,256	8,939,666
Physical health				
Congenital abnormality	1.44 [1.31,1.57]	0.64 [0.52,0.77]	0.45 [0.31,0.6]	0.98 [0.90,1.06]
Asthma	0.36 [0.18,0.55]	-0.28 [-0.49,-0.06]	1.57 [1.30,1.85]	0.39 [0.25,0.52]
Obesity	0.00 [-0.0,0.08]	0.46 [0.32,0.61]	1.12 [0.91,1.33]	0.40 [0.33,0.48]
Hearing problem	1.23 [1.13,1.33]	0.70 [0.60,0.80]	0.59 [0.48,0.69]	0.92 [0.86,0.99]
Vision problem	1.37 [1.25,1.49]	1.44 [1.30,1.58]	1.22 [1.07,1.36]	1.35 [1.27,1.43]
Middle ear infection	2.38 [2.13,2.63]	-0.27 [-0.47,-0.07]	0.20 [0.01,0.40]	1.19 [1.05,1.33]
Respiratory infection	1.67 [1.36,1.97]	-1.00 [-1.4,-0.70]	0.99 [0.58,1.39]	0.72 [0.51,0.93]
Dental problem	2.31 [2.14,2.47]	1.81 [1.64,1.98]	2.00 [1.80,2.20]	2.16 [2.06,2.27]
Dermatological problem	1.99 [1.77,2.20]	1.39 [1.15,1.64]	3.36 [3.02,3.70]	2.19 [2.04,2.34]
Injuries	2.23 [2.01,2.44]	1.95 [1.67,2.23]	7.93 [7.53,8.34]	3.45 [3.29,3.62]
Complex chronic condition	1.13 [0.99,1.27]	1.31 [1.13,1.49]	3.09 [2.81,3.37]	1.67 [1.56,1.79]
Developmental disorder				
Autism/PDD	0.92 [0.81,1.04]	0.96 [0.82,1.10]	1.05 [0.88,1.21]	0.97 [0.88,1.05]
Developmental Delay	7.70 [7.43,7.97]	2.79 [2.53,3.04]	1.88 [1.66,2.11]	4.91 [4.74,5.07]
Mental health disorder				
Depression	1.14 [1.06,1.21]	6.01 [5.78,6.23]	16.1 [15.7,16.5]	6.05 [5.91,6.19]
Anxiety	2.81 [2.67,2.95]	5.21 [4.98,5.45]	8.75 [8.39,9.11]	4.84 [4.71,4.98]
Trauma and Stress	24.7 [24.4,25.0]	31.9 [31.5,32.3]	27.0 [26.5,27.4]	27.4 [27.2,27.6]
ADHD/Conduct/Impulse	9.83 [9.57,10.0]	15.8 [15.4,16.2]	21.1 [20.7,21.6]	14.0 [13.7,14.2]
Suicidality/Self-Harm	0.29 [0.26,0.33]	2.08 [1.96,2.20]	5.68 [5.43,5.92]	2.05 [1.98,2.13]
Substance use-related disorder	0.18 [0.15,0.21]	0.59 [0.53,0.66]	11.6 [11.2,11.9]	2.96 [2.88,3.05]
Alcohol	0.06 [0.04,0.08]	0.14 [0.11,0.17]	2.41 [2.25,2.57]	0.62 [0.58,0.66]
Tobacco	-0.00 [-0.0,0.00]	0.06 [0.04,0.08]	2.33 [2.17,2.49]	0.57 [0.53,0.61]
Drug	0.12 [0.09,0.15]	0.43 [0.38,0.48]	10.3 [9.98,10.6]	2.55 [2.47,2.63]

Notes: Abbreviations: PDD, pervasive developmental disorder; ADHD, attention-deficit/hyperactivity disorder

[a] Medicaid claims from the 2014-2020 Transformed Medicaid Statistical Information System Analytic Files (TAF) and the 2014-2015 Medicaid Analytical eXtract (MAX). The unit of observation are person-years (N = 8,939,666), from 1,985,180 children aged 4-18 experiencing POUD (identified with procedure, diagnostic, and NDC codes).

[b] The sample of person-years was stratified by foster care involvement, identified using Medicaid eligibility, procedure and diagnostic codes.

[c] Linear regression was used to compute adjusted differences in outcome prevalence across person-years with and without foster care involvement for each age group. Regressions controlled for demographics and enrollment days in Table 1, and for state and year fixed-effects. Standard errors were clustered at the person-level.

© 2024 Meinhofer A et al. *JAMA Network Open.*

eTable 7. Mean Differences in Health Care Utilization by Foster Care Status[a,b,c]

	Adjusted Mean Differences [95% CI]			
Age group	4 to 8	9 to 13	14 to 18	Total
Person Years (PY)	3,469,667	3,131,743	2,338,256	8,939,666
Primary Care and Prevention				
Well-Child Visit	18.9 [18.6,19.1]	22.5 [22.1,22.8]	20.7 [20.3,21.2]	20.6 [20.4,20.8]
Immunization	11.1 [10.8,11.4]	10.7 [10.3,11.0]	10.0 [9.61,10.4]	10.8 [10.6,11.0]
Vision Exam	5.21 [4.96,5.46]	7.11 [6.80,7.42]	6.59 [6.24,6.94]	6.10 [5.93,6.28]
Hearing Exam	6.81 [6.56,7.07]	8.01 [7.71,8.31]	7.55 [7.22,7.89]	7.32 [7.14,7.49]
Laboratory Work	8.01 [7.73,8.29]	8.69 [8.35,9.03]	15.5 [15.1,16.0]	10.1 [9.95,10.3]
Developmental Screen	3.91 [3.72,4.11]	1.99 [1.82,2.15]	1.83 [1.65,2.01]	2.91 [2.80,3.03]
Metabolic Screen	0.92 [0.83,1.01]	3.77 [3.56,3.98]	7.26 [6.94,7.58]	3.24 [3.13,3.36]
Lead Screen	4.67 [4.49,4.85]	0.98 [0.90,1.06]	0.75 [0.67,0.84]	2.64 [2.55,2.73]
STI Screen	1.13 [1.06,1.21]	1.58 [1.48,1.69]	10.5 [10.1,10.8]	3.63 [3.53,3.74]
Health Counseling	3.30 [3.12,3.49]	3.90 [3.69,4.12]	4.80 [4.53,5.07]	3.90 [3.77,4.02]
Dental Services	18.7 [18.4,19.0]	19.1 [18.7,19.4]	20.3 [19.8,20.7]	19.0 [18.8,19.2]
Behavioral Services				
Mental Health Screen	8.42 [8.22,8.62]	11.4 [11.1,11.7]	11.4 [11.0,11.8]	9.98 [9.82,10.1]
Mental Health Provider	26.1 [25.7,26.4]	32.8 [32.4,33.2]	34.5 [34.0,35.0]	29.9 [29.7,30.1]
Other Healthcare Settings				
Inpatient Hospital	2.17 [2.01,2.33]	4.19 [3.97,4.40]	7.43 [7.11,7.76]	4.05 [3.92,4.18]
Emergency Room	0.04 [-0.2,0.32]	2.77 [2.43,3.11]	10.8 [10.4,11.3]	3.53 [3.32,3.73]
Urgent Care Clinic	0.80 [0.58,1.01]	-0.11 [-0.3,0.12]	1.42 [1.12,1.72]	0.70 [0.55,0.84]
School-Based Services	6.12 [5.87,6.38]	7.67 [7.34,7.99]	7.47 [7.12,7.82]	6.79 [6.61,6.98]

Notes: Abbreviations: PDD, pervasive developmental disorder; ADHD, attention-deficit/hyperactivity disorder
[a] Medicaid claims from the 2014-2020 Transformed Medicaid Statistical Information System Analytic Files (TAF) and the 2014-2015 Medicaid Analytical eXtract (MAX). The unit of observation are person-years (N = 8,939,666), from 1,985,180 children aged 4-18 experiencing POUD (identified with procedure, diagnostic, and NDC codes).
[b] The sample of person-years was stratified by foster care involvement, identified using Medicaid eligibility, procedure and diagnostic codes.
[c] Linear regression was used to compute adjusted differences in outcome prevalence across person-years with and without foster care involvement for each age group. Regressions controlled for demographics and enrollment days in Table 1, and for state and year fixed-effects. Standard errors were clustered at the person-level.

© 2024 Meinhofer A et al. *JAMA Network Open.*

eReferences

1. Geen R, Sommers A, Cohen M, Urban Institute. Medicaid Spending on Foster Children. 2005; https://webarchive.urban.org/UploadedPDF/311221_medicaid_spending.pdf. Accessed August 1, 2023.
2. Raghavan R, Aarons GA, Roesch SC, Leslie LK. Longitudinal patterns of health insurance coverage among a national sample of children in the child welfare system. *Am J Public Health.* 2008;98(3):478-484.
3. Child Welfare Information Gateway. Health-Care Coverage for Children and Youth in Foster Care – and After. 2022; https://www.childwelfare.gov/pubpdfs/health_care_foster.pdf. Accessed August 1, 2023.
4. Stoltzfus E, Congressional Research Service. A Detailed Overview of Program Eligibility and Funding for Foster Care, Adoption Assistance and Kinship Guardianship Assistance under Title IV-E of the Social Security Act. 2012; https://crsreports.congress.gov/product/details?prodcode=R42792. Accessed August 1, 2023.
5. Schneider A, Gantner L, Maag I, Borst MM, Wensing M, Szecsenyi J. Are ICD-10 codes appropriate for performance assessment in asthma and COPD in general practice? Results of a cross sectional observational study. *BMC Health Serv Res.* 2005;5(1):11.
6. Juhn Y, Kung A, Voigt R, Johnson S. Characterisation of children's asthma status by ICD-9 code and criteria-based medical record review. *Prim Care Respir J.* 2011;20(1):79-83.
7. Ndibazza J, Lule S, Nampijja M, et al. A description of congenital anomalies among infants in Entebbe, Uganda. *Birth Defects Res A Clin Mol Teratol.* 2011;91(9):857-861.
8. Holmes LB, Westgate MN. Using ICD-9 codes to establish prevalence of malformations in newborn infants. *Birth Defects Res A Clin Mol Teratol.* 2012;94(4):208-214.
9. Feudtner C, Feinstein JA, Zhong W, Hall M, Dai D. Pediatric complex chronic conditions classification system version 2: updated for ICD-10 and complex medical technology dependence and transplantation. *BMC Pediatr.* 2014;14(1):199.
10. Gribsholt SB, Pedersen L, Richelsen B, Thomsen RW. Validity of ICD-10 diagnoses of overweight and obesity in Danish hospitals. *Clin Epidemiol.* 2019;11:845-854.
11. Mocarski M, Tian Y, Smolarz BG, McAna J, Crawford A. Use of International Classification of Diseases, Ninth Revision Codes for Obesity: Trends in the United States from an Electronic Health Record-Derived Database. *Popul Health Manag.* 2018;21(3):222-230.
12. Osler M, Christensen GT, Mortensen EL, Christensen K, Garde E, Rozing MP. Hearing loss, cognitive ability, and dementia in men age 19-78 years. *Eur J Epidemiol.* 2019;34(2):125-130.
13. Miyagishima R, Hopper T, Hodgetts B, Soos B, Williamson T, Drummond N. Development of a case definition for hearing loss in community-based older adults: a cross-sectional validation study. *CMAJ Open.* 2021;9(3):E796-E801.
14. van Duijnhoven J, Aarts M, Aries M, Rosemann A, Kort H. Systematic review on the interaction between office light conditions and occupational health: Elucidating gaps and methodological issues. *Indoor Built Environ.* 2017;28(2):152-174.
15. Ganz ML, Xuan Z, Hunter DG. Prevalence and correlates of children's diagnosed eye and vision conditions. *Ophthalmology.* 2006;113(12):2298-2306.
16. Figueiredo RL, Singhal S, Dempster L, Hwang SW, Quinonez C. The accuracy of International Classification of Diseases coding for dental problems not associated with trauma in a hospital emergency department. *J Public Health Dent.* 2015;75(4):343-347.
17. Allareddy V, Kim MK, Kim S, Gajendrareddy P, Karimbux NY, Nalliah RP. Hospitalizations primarily attributed to dental conditions in the United States in 2008. *Oral Surg Oral Med Oral Pathol Oral Radiol.* 2012;114(3):333-337.
18. Samannodi M. Hospital Admissions Related to Infections and Disorders of the Skin and Subcutaneous Tissue in England and Wales. *Healthcare (Basel).* 2022;10(10):2028.

© 2024 Meinhofer A et al. *JAMA Network Open.*

19. Peñate Y, Borrego L, Hernández N, Islas D. Pediatric dermatology consultations: a retrospective analysis of inpatient consultations referred to the dermatology service. *Pediatr Dermatol.* 2012;29(1):115-118.
20. Horsky J, Drucker EA, Ramelson HZ. Accuracy and Completeness of Clinical Coding Using ICD-10 for Ambulatory Visits. *AMIA Annu Symp Proc.* 2017;2017:912-920.
21. Adams DJ, Susi A, Erdie-Lalena CR, et al. Otitis Media and Related Complications Among Children with Autism Spectrum Disorders. *J Autism Dev Disord.* 2016;46(5):1636-1642.
22. Chicaiza-Ayala W, Henríquez-Trujillo AR, Ortiz-Prado E, Douce RW, Coral-Almeida M. The burden of acute respiratory infections in Ecuador 2011-2015. *PLoS One.* 2018;13(5):e0196650.
23. Carpenter DO, Ma J, Lessner L. Asthma and infectious respiratory disease in relation to residence near hazardous waste sites. *Ann N Y Acad Sci.* 2008;1140:201-208.
24. Gil A, Carrasco P, Jiménez R, San-Martín M, Oyagüez I, González A. Burden of hospitalizations attributable to rotavirus infection in children in Spain, period 1999-2000. *Vaccine.* 2004;22(17-18):2221-2225.
25. Lynen Jansen P, Stallmach A, Lohse A, Lerch M. Development of gastrointestinal infectious diseases between 2000 and 2012. *Z Gastroenterol.* 2014;52(6):549-557.
26. Clark D, Black A, Skavdahl D, Hallagan L. Open-access programs for injury categorization using ICD-9 or ICD-10. *Inj Epidemiol.* 2018;5(1):11.
27. Meinhofer A, Hinde JM, Keyes KM, Lugo-Candelas C. Association of Comorbid Behavioral and Medical Conditions With Cannabis Use Disorder in Pregnancy. *JAMA Psychiatry.* 2022;79(1):50-58.
28. Kim GE, Jo MW, Shin YW. Increased prevalence of depression in South Korea from 2002 to 2013. *Sci Rep.* 2020;10(1):16979.
29. Chen MH, Pan TL, Bai YM, et al. Postpartum Depression and Psychosis and Subsequent Severe Mental Illnesses in Mothers and Neurodevelopmental Disorders in Children: A Nationwide Study. *J Clin Psychiatry.* 2021;82(4).
30. Li X, Sundquist J, Sundquist K. Sibling risk of anxiety disorders based on hospitalizations in Sweden. *Psychiatry Clin Neurosci.* 2011;65(3):233-238.
31. Carballo JJ, Baca-Garcia E, Blanco C, et al. Stability of childhood anxiety disorder diagnoses: a follow-up naturalistic study in psychiatric care. *Eur Child Adolesc Psychiatry.* 2010;19(4):395-403.
32. Stewart C, Crawford PM, Simon GE. Changes in Coding of Suicide Attempts or Self-Harm With Transition From ICD-9 to ICD-10. *Psychiatr Serv.* 2017;68(3):215.
33. Simon GE, Shortreed SM, Boggs JM, et al. Accuracy of ICD-10-CM encounter diagnoses from health records for identifying self-harm events. *J Am Med Inform Assoc.* 2022;29(12):2023-2031.
34. Møller L, Augsburger M, Elklit A, Søgaard U, Simonsen E. Traumatic experiences, ICD-11 PTSD, ICD-11 complex PTSD, and the overlap with ICD-10 diagnoses. *Acta Psychiatr Scand.* 2020;141(5):421-431.
35. Gravely AA, Cutting A, Nugent S, Grill J, Carlson K, Spoont M. Validity of PTSD diagnoses in VA administrative data: comparison of VA administrative PTSD diagnoses to self-reported PTSD Checklist scores. *J Rehabil Res Dev.* 2011;48(1):21-30.
36. Steinhausen HC, Erdin A. A comparison of ICD-9 and ICD-10 diagnoses of child and adolescent psychiatric disorders. *J Child Psychol Psychiatry.* 1991;32(6):909-920.
37. Lee SI, Schachar RJ, Chen SX, et al. Predictive validity of DSM-IV and ICD-10 criteria for ADHD and hyperkinetic disorder. *J Child Psychol Psychiatry.* 2008;49(1):70-78.
38. Gruschow SM, Yerys BE, Power TJ, Durbin DR, Curry AE. Validation of the Use of Electronic Health Records for Classification of ADHD Status. *J Atten Disord.* 2019;23(13):1647-1655.
39. Erskine HE, Ferrari AJ, Nelson P, et al. Epidemiological modelling of attention-deficit/hyperactivity disorder and conduct disorder for the Global Burden of Disease Study 2010. *J Child Psychol Psychiatry.* 2013;54(12):1263-1274.

© 2024 Meinhofer A et al. *JAMA Network Open.*

40. Blanz B, Schmidt M, Esser N. Conduct disorders (CD): the reliability and validity of the new ICD-10-categories. *Acta Paedopsychiatr.* 1990;53(2):93-103.
41. Møller LR, Sørensen MJ, Thomsen PH. ICD-10 classification in Danish child and adolescent psychiatry--have diagnoses changed after the introduction of ICD-10? *Nord J Psychiatry.* 2007;61(1):71-78.
42. Hong M, Moon DS, Chang H, et al. Incidence and Comorbidity of Reactive Attachment Disorder: Based on National Health Insurance Claims Data, 2010-2012 in Korea. *Psychiatry Investig.* 2018;15(2):118-123.
43. Blanz B, Amorosa H, Schmidt MH. Psychiatric disorders in children and adolescents: results of the ICD-10 field trial. *Pharmacopsychiatry.* 1990;23(S 4):173-176.
44. McCabe RJ, Rothery DJ, Wrate RM, Aspin J, Bryce JG. Diagnosis in adolescent inpatients: diagnostic confidence and comparison of diagnoses using ICD-9 and DSM-III. *Eur Child Adolesc Psychiatry.* 1996;5(3):147-154.
45. Dodds L, Spencer A, Shea S, et al. Validity of autism diagnoses using administrative health data. *Chronic Dis Can.* 2009;29(3):102-107.
46. Volkmar FR, Cicchetti DV, Bregman J, Cohen DJ. Three diagnostic systems for autism: DSM-III, DSM-III-R, and ICD-10. *J Autism Dev Disord.* 1992;22(4):483-492.
47. Lingren T, Chen P, Bochenek J, et al. Electronic Health Record Based Algorithm to Identify Patients with Autism Spectrum Disorder. *PLoS One.* 2016;11(7):e0159621.
48. Lehti V, Gyllenberg D, Suominen A, Sourander A. Finnish-born children of immigrants are more likely to be diagnosed with developmental disorders related to speech and language, academic skills and coordination. *Acta Paediatr.* 2018;107(8):1409-1417.
49. Mann JR, Crawford S, Wilson L, McDermott S. Does race influence age of diagnosis for children with developmental delay? *Disabil Health J.* 2008;1(3):157-162.
50. American Academy of Pediatrics. Coding for Pediatric Preventive Care 2022. 2022; https://downloads.aap.org/AAP/PDF/Coding%20Preventive%20Care.pdf. Accessed August 1, 2023.

© 2024 Meinhofer A et al. *JAMA Network Open.*
