# Supplementary material for: Foster Care and Health in Medicaid-Enrolled Children Experiencing Parental Opioid Use Disorder
Source: JAMA Netw Open. 2024 May 8;7(5):e2410432. doi: 10.1001/jamanetworkopen.2024.10432 (PMC11079692; doi:10.1001/jamanetworkopen.2024.10432)
Supplement: Supplement 3. — Data Sharing Statement [file jamanetwopen-e2410432-s003.pdf]

Data Sharing Statement

Meinhofer. Foster Care and Health in Medicaid-Enrolled Children Experiencing Parental Opioid Use Disorder. *JAMA Netw Open*. Published May 08, 2024.
doi:10.1001/jamanetworkopen.2024.10432

Data
Data available: No

Additional Information
Explanation for why data not available: Data use agreement with CMS restricts our ability to share Medicaid claims data.
